# Markers of diuretic resistance in emergency department patients with acute heart failure

**DOI:** 10.1186/s12245-017-0143-x

**Published:** 2017-05-08

**Authors:** Andrew Doering, Cathy A. Jenkins, Alan B. Storrow, JoAnn Lindenfeld, Gregory J. Fermann, Karen F. Miller, Matthew Sperling, Sean P. Collins

**Affiliations:** 1 Department of Emergency Medicine, Vanderbilt University Medical Center, Nashville, TN USA; 2 Department of Biostatistics, Vanderbilt University Medical Center, Nashville, TN USA; 3 Department of Internal Medicine, Division of Cardiology, Vanderbilt University Medical Center, Nashville, TN USA; 4 Department of Emergency Medicine, University of Cincinnati, Cincinnati, OH USA

**Keywords:** Emergency department, Acute heart failure, Diuretic resistance

## Abstract

**Background:**

Loop diuretics are common therapy for emergency department (ED) patients with acute heart failure (AHF). Diuretic resistance (DR) is a term used to describe blunted natriuretic response to loop diuretics. It would be important to detect DR prior to it becoming clinically apparent, so early interventions can be initiated. However, several definitions have been proposed, and it is not clear if they identify similar patients. We compared these definitions and described the clinical characteristics of patients who fulfilled them.

**Methods:**

To qualify for this secondary analysis of 1033 ED patients with AHF, all patients needed to receive intravenous diuretics in the ED and have urine available within 24 h of their ED evaluation. A poor diuretic response, suggesting DR, was characterized by (1) a fractional sodium excretion (FeNa) of less than 0.2%; (2) spot urinary sodium of less than 50 meq/L; and (3) a urinary Na/K ratio <1.0. McNemar’s test was used to compare the different cohorts identified by the three definitions. Secondary analyses evaluated associations between each DR definition and hospital length of stay (LOS), ED revisits and rehospitalizations for AHF, and mortality using the Wilcoxon rank-sum tests and linear regression or Pearson chi-square test and logistic regression, as appropriate.

**Results:**

The median age of the 187 patients was 64, and 50% were African-American. There were 5.9% of patients with a FeNa less than 0.2%, 17.1% had urinary sodium less than 50 meq/L, and 10.7% had a urinary Na/K ratio <1.0. The three definitions identified significantly different patients with very little overlap (*p* < 0.02 for all comparisons). There were 37 (19.8%) patients who were readmitted to the ED or hospital or died within 30 days of ED evaluation. Patients with spot urinary sodium less than 50 meq/L were more likely to be readmitted (*p* = 0.03).

**Conclusions:**

The patient proportion with poor natriuresis and DR varies depending on the definition used. Early ED therapy would be impacted at different rates if clinical decisions are made based on these definitions. These findings need to be further explored in a prospective ED-based study.

**Trial registration:**

ClinicalTrials.gov, NCT00508638

**Electronic supplementary material:**

The online version of this article (doi:10.1186/s12245-017-0143-x) contains supplementary material, which is available to authorized users.

## Background

Over the last decade, there have been nearly one million annual emergency department (ED) visits for acute heart failure (AHF) [1]. Intravenous loop diuretics are commonly administered to promote diuresis, natriuresis, and congestion relief in these patients [2, 3]. While diuretics often lead to symptomatic improvement, they have not resulted in decreases in mortality or hospital readmission, and some studies have found that their use in hospitalized patients has been associated with detrimental outcomes [4].

In AHF, decreased cardiac output leads to arterial underfilling, increased renin-angiotensin-aldosterone system activity, and increased proximal tubule sodium reabsorption [5]. Retention of sodium and water due to inadequate natriuresis and diuresis are hallmarks of heart failure (HF). Patients with HF have a markedly reduced rate of renal sodium excretion, and cumulative sodium retention has been closely correlated with an increase in body weight [6]. Loop diuretics are administered to inhibit sodium reabsorption in the thick ascending limb of the loop of Henle and are intended to increase natriuresis. When loop diuretics are effective, the urinary sodium rises and the urinary potassium falls. Poor gut absorption of orally administered loop diuretics, decreased delivery to molecular targets, low serum albumin, renal tubular hypertrophy, and circulating organic acids inhibiting the organic anion transporter all lead to a diminished effect of diuretics, resulting in impaired urinary sodium excretion [7]. Even when diuretics are given intravenously, diuretic “braking” can be encountered, negating intended natriuresis and contributing to diuretic resistance in patients with AHF. [7]

Furthermore, a subset of HF patients with a decreased response to diuretic therapy are termed “diuretic resistant” and have a blunted natriuretic response, though the frequency of this phenomenon is not well established [7]. Inadequate natriuresis and diuresis in patients who develop diuretic resistance have been associated with prolonged hospital lengths of stay and increased mortality [8, 9]. While clinical characteristics of patients with diuretic resistance have been identified, a reliable prospective measure identifying those who have suboptimal diuretic and natriuretic responsiveness has not been extensively studied [10]. The inability to predict diuretic responsiveness may delay intensified therapy, thus prolonging hospitalization and delaying decongestion.

Until recently, investigations had focused on defining diuretic and natriuretic responsiveness based on change in body weight or urine output relative to the dose of furosemide administered [8, 9, 11, 12]. These definitions require up to 96 h to identify diuretic resistance, do not allow a real-time measure of response to diuretic therapy, and have not been conducted in the ED [10]. Without an early definition of diuretic and natriuretic responsiveness, it is difficult to design prospective ED-based studies of alternative therapies to loop diuretics, such as diuretic combinations, natriuretic doses of mineralocorticoid receptor antagonists, vasoactive medications, and ultrafiltration.

The purpose of the study was not to determine the ideal definition for diuretic resistance but to better understand how the different definitions compare. Definitions generally utilize inexpensive and rapidly available urine electrolytes. The fractional sodium excretion (FeNa) is the percentage of sodium filtered by the kidney and excreted in the urine; it has been used to assess natriuresis in HF patients [13–15]. Baseline FeNa is reduced to less than 1.0% in patients with HF, and a baseline FeNa of less than 0.2% is associated with poor natriuretic response [7, 13–15]. Further, in liver cirrhosis, a condition also associated with poor natriuretic response to diuretics, a natriuresis lower than 50 meq in 8 h after a single intravenous dose of loop diuretics was a predictor of refractory ascites and has been used to define poor natriuretic responsiveness in this population [16]. An exploratory definition of a urine Na/K ratio <1.0 was included based on our suspicion that patients with poor natriuretic responsiveness would have disproportionately low ratios of urinary sodium relative to potassium. Understanding the strengths, limitations, and biases of these observations is fundamental to defining diuretic resistance. To better understand these concepts, we directly compared definitions of diuretic resistance in a cohort enrolled in a prospective observational study in ED patients with AHF. A secondary aim was to evaluate hospital length of stay (LOS), 30-day mortality, repeat ED visits, and rehospitalizations for AHF in patients who fulfilled each of the definitions.

## Methods

### Patients

This study was approved by the Vanderbilt University Medical Center Institutional Review Board as exempt from review (IRB# 150508). Our subjects consisted of a subset of patients with AHF enrolled in a prospective ED cohort study [2, 17]. Eligible patients received at least one dose of intravenous diuretics and were admitted to the hospital with a diagnosis of AHF. Urine was collected within 12–24 h of their ED visit and analyzed for potassium, sodium, and creatinine. Patients were contacted by telephone and had a medical record review performed to determine 30-day outcomes in the prior cohort study.

### Measures of diuretic resistance

For our analysis, diuretic resistance was defined using three separate criteria: (1) a baseline FeNa of less than 0.2% [7, 13–15]; (2) a spot urinary sodium of less than 50 meq/L at 12–24-h urine measurement [16]; and (3) a spot urine Na/K ratio <1.0.

### Statistical analysis

Descriptive statistics, including median and percentages, were used to describe the study population and time of diuretic administration to acquisition of urine. To assess agreement between the definitions of diuretic resistance, we used Cohen’s Kappa coefficient and McNemar’s test. Wilcoxon rank-sum tests and linear regression were used to assess the association of the biomarkers for diuretic resistance with hospital LOS; Pearson chi-square tests and logistic regression were used to assess the association of the diuretic resistance with AHF-related ED revisits, rehospitalizations for AHF, and mortality within 30 days of the index ED visit. For all regressions, the biomarker was kept continuous and fit with restricted cubic splines (three knots) to relax the linearity assumption.

## Results

Our study included 187 patients (Table 1). Sixty-five percent were male, and the median age was 64 years (interquartile range (IQR) 55, 73). The median systolic blood pressure (SBP) was 149 mmHg (IQR = 130, 178), and 50% were African-American. The median blood urea nitrogen and creatinine in our cohort were 22 mg/dL (IQR = 15, 35) and 1.4 mg/dL (IQR = 1.1, 2.1), respectively. The median b-type natriuretic peptide (BNP) was 1232 pg/mL (IQR = 545, 2198), and 33% had an ejection fraction (EF) greater than 55% on echocardiogram. The median time from diuretic administration to collection of urine for electrolyte measurement was 8.3 h (IQR = 3.0, 12.9).

**Table 1:** Descriptive statistics on the entire cohort

	*N* = 187	Median or %	Quartiles or frequencies
Age	187	64	(55, 73)
Sex	187		
Female		35%	(66)
Male		65%	(121)
Race	187		
AA		50%	(93)
Other		50%	(94)
History of renal disease	184	26%	47
Home diuretic dose (mg)	77	60	(40, 80)
SBP (mmHg)	187	149	(130, 178)
BUN	185	22	(15, 35)
Serum creatinine (baseline)	187	1.4	(1.1, 2.1)
Serum creatinine (12–24 h)	187	1.5	(1.1, 2.2)
Urine creatinine (12–24 h)	187	42	(23, 70)
eGFR	187	53	(32, 72)
BNP (pg/mL)	187	1232	(545, 2198)
Urinary sodium (12–24 h)	187	90	(65, 110)
Na/K ratio	187	3.8	(1.8, 6.4)
Serum sodium (baseline)	187	140	(138, 142)
Serum sodium (12–24 h)	187	139	(138, 141)
FeNa	187	2.4	(1.0, 5.1)
Ejection fraction	175		
Normal (greater than 55%)		33%	(57)
Mild (45–55%)		14%	(24)
Moderate (25–44%)		23%	(41)
Severe (less than 25%)		30%	(53)
ED lasix/furosemide dose categorized	159		
≥80 mg		33%	(52)
<80 mg		67%	(107)
Hours from diuretic (first of non-initial dose in ED) to lab draw at second visit^c^	132	8.3	(3.0, 12.9)
LOS (days)	187	4	(2, 6)
Urine output up to second visit	172	1750	(829, 2759)
ED revisit for HF	187		
No		86%	(161)
Yes		14%	(26)
Readmission for HF	187		
No		84%	(158)
Yes		16%	(29)
Status	187		
Alive		96%	(179)
Deceased		4%	(8)

^a^Median for continuous variables or percent for dichotomous variables

^b^Lower and upper quartile for continuous variables or frequencies

^c^Only 132 patients had the time window recorded from diuretic dose to second lab draw

### Measures of natriuretic responsiveness

We identified 11 (5.9%) patients with diuretic resistance based on a FeNa of <0.2% (Additional file 1). These subjects, compared to those without resistance, had similar ED-measured SBP (142 vs 149 mmHg, respectively, *p* = 0.24) and BNP (894 vs 1249 pg/mL, *p* = 0.14) but were more likely to have better renal function (serum Cr 1.0 vs 1.4 mg/dL, *p* = 0.007). There were 32 (17.1%) patients defined as diuretic resistant based on spot urinary sodium value <50 meq/L. Using this definition, diuretic resistant and non-diuretic resistant patients had similar ED-measured renal function (serum Cr 1.4 vs 1.4 mg/dL, respectively, *p* = 0.57), SBP (147 vs 149 mmHg, respectively, *p* = 0.79), and BNP levels (1468 vs 1217 pg/mL, *p* = 0.15). Despite approximately one-third of both groups receiving 80 mg or greater of intravenous furosemide, the median urinary output 12–24 h later in those who fulfilled this definition was nearly 900 mL less than those who did not (*p* = 0.005) (Table 2). Twenty (10.7%) patients were diuretic resistant using a urinary Na/K ratio of <1.0 (Additional file 2). Patients with and without diuretic resistance based on this definition had similar baseline characteristics, but those of AA race (25 vs 53%, *p* = 0.019) were much less likely to be diuretic resistant.

**Table 2:** Descriptive statistics by diuretic resistance assessed using spot urinary sodium cut point of 50 meq/L

	Number	Normal (*N* = 155)		Diuretic resistant (*N* = 32)		*p* value
Age	187	63	(54, 73)	68	(62, 77)	0.031
Sex	187					0.62
Female		36%	56	31%	10	
Male		64%	99	69%	22	
Race	187					0.0562
AA		54%	82	34%	11	
Other		47%	73	66%	21	
History of renal disease	184					0.33
No		73%	111	81%	26	
Yes		27%	41	19%	6	
Home diuretic dose (mg)	77	60	(40, 80)	60	(40, 80)	0.78
SBP	187	149	(130, 178)	147	(129, 174)	0.791
BUN	185	21	(15, 35)	27	(17, 38)	0.161
Serum creatinine (baseline)	187	1.4	(1.0, 2.0)	1.4	(1.1, 2.1)	0.571
Serum creatinine (12–24 h)	187	1.5	(1.1, 2.2)	1.4	(1.2, 2.1)	0.721
Urine creatinine (12–24 h)	187	35	(22, 59)	75 (46, 124)	75 (46, 124)	<0.0011
eGFR	187	53	(33, 73)	51 (30, 67)	51 (30, 67)	0.361
BNP	187	1217	(532, 2100)	1468	(864, 2866)	0.151
Urinary sodium (12–24 h)	187	99	(82, 114)	33	(23, 41)	<0.0011
Na/K ratio	187	4.5	(2.59, 6.79)	0.8	(0.49, 1.41)	<0.0011
Serum sodium (baseline)	187	140	(138, 142)	139	(138, 140)	0.111
Serum sodium (12–24 h)	187	139	(138, 141)	138	(137, 140)	0.151
FeNa	187	3.35	(1.52, 5.65)	0.73	(0.18, 1.12)	<0.0011
Ejection fraction	175					0.632
Normal (greater than 55%)		31%	(45)	40%	(12)	
Mild (45–55%)		14%	(21)	10%	(3)	
Moderate (25–44%)		23%	(33)	27%	(8)	
Severe (less than 25%)		32%	(46)	23%	(7)	
ED lasix/furosemide dose categorized	159					0.822
≥80 mg		32%	(43)	35%	(9)	
<80 mg		68%	(90)	65%	(17)	
LOS (days)	187	3	(2, 6)	4	(3, 7)	0.221
Urine output up to second visit	172	1925	(1060, 3000)	1050	(690, 1925)	0.0051
ED revisit for HF	187					0.382
No		87%	(135)	81%	(26)	
Yes		13%	(20)	19%	(6)	
Readmission for HF	187					0.032
No		87%	(135)	72%	(23)	
Yes		13%	(20)	28%	(9)	
Status	187					0.122
Alive		97%	(150)	91%	(29)	
Deceased		3%	(5)	9%	(3)	

A comparison between our three definitions of diuretic resistance (Tables 3, 4, and 5) found each identified a significantly different (*p* ≤ 0.02) patient cohort. The Kappa for agreement between FeNa and measured urinary sodium was 0.41 (95% CI 0.22–0.6). For FeNa and urine Na/K ratio, the Kappa was 0.62 (95% CI 0.41–0.82). Finally, for urine Na/K ratio and measured urinary sodium concentration, the Kappa was 0.60 (95% CI 0.44–0.77)

**Table 3:** Comparison of measured urinary sodium and FeNa as measures of diuretic resistance

		Measured urinary sodium		
		Normal	Diuretic resistant	
FeNa	Normal	154	22	*p* < 0.001
	Diuretic resistant	1	10

**Table 4:** Comparison of Na/K ratio and FeNa as measures of diuretic resistance

		Na/K ratio		
		Normal	Diuretic resistant	
FeNa	Normal	166	10	*p* = 0.02
	Diuretic resistant	1	10

**Table 5:** Comparison of predicted urinary sodium and measured urinary sodium as measures of diuretic resistance

		Measured urinary sodium		
		Normal	Diuretic resistant	
Na/K ratio	Normal	152	15	*p* = 0.01
	Diuretic resistant	3	17	

### Hospital LOS, AHF revisits, AHF readmission, and mortality

**Fig. 1:**
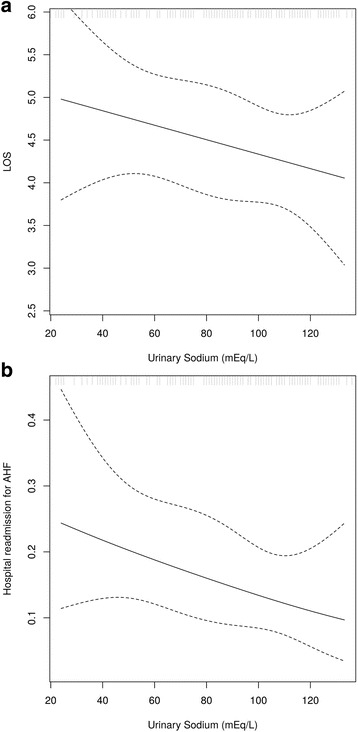
**a** Spot urinary sodium vs hospital LOS in days. **b** Spot urinary sodium vs likelihood of hospital readmission

There was no difference in hospital LOS, ED revisits or hospital readmissions for AHF, or death between those who did and did not fulfill the definition of diuretic resistance based on FeNa <0.2%. Patients who were diuretic resistant based on a spot urinary Na <50 meq/L had a higher rate of hospital readmission for AHF compared to those who were not (28 vs 13%, *p* = 0.03). While diuretic resistance as defined by the dichotomized urinary Na <50 meq/L failed to show significant associations with LOS, predicted LOS and hospital readmission from the linear regression suggested decreasing LOS and hospital readmission with increasing urinary sodium. (Table 2, Fig. 1a, b). Patients fulfilling the third criterion standard for diuretic resistance, a urine Na/K ratio of <1, had no differences in LOS, 30-day ED revisit, or hospital readmission for AHF versus those with Na/K ratios of >1.

## Discussion

We directly compared three definitions of diuretic resistance in 187 ED patients admitted for AHF. Our investigation is the only study to date performed in ED patients after their first dose of diuretic, as other studies focused on inpatients who had received multiple doses of diuretics. Our three definitions of diuretic resistance identified distinctly different patient cohorts, and the prevalence varied widely based on these definitions. Diuretic resistance in our population ranged from 5.9% using FeNa <0.2 to 17.1% using spot urinary Na <50 meq/L. This is an important finding as we seek to identify diuretic resistance earlier in a patient’s course; the definition utilized would impact the proportion of patients identified. Those who fulfilled a definition of diuretic resistance based on urine Na <50 meq/L were more likely to have an increased hospital LOS and an increased proportion of AHF readmissions. This has been seen in prior inpatient studies as well [9, 18].

Traditional definitions have required greater than 24 h to classify patients as diuretic resistant by using change in weight divided by milligram equivalent of loop diuretic [9, 19]. A formula was recently derived to quantify the relationship between early measures of urinary sodium and 6-h natriuresis [10]. However, these patients were enrolled a median of 4 days after hospital admission and would not be reflective of the more acute, early diuresis seen in an ED AHF population. The timeframe for definitions we investigated was less than 24 h from ED presentation.

Early identification of ED patients with AHF who will have a suboptimal response to traditional diuretic dosing could help guide therapy and may have implications regarding hospital LOS, readmission, and the cost of care. Those patients identified early in their ED stay as non-responders could be given a higher dose of loop diuretic, a thiazide, aldosterone antagonist, or vasoactive therapy as a means of decongestive therapy. However, other factors need to be considered when evaluating readmission, such as dietary and medication non-adherence. Further, identification of diuretic resistance was not consistent across the investigated definitions, highlighting the importance of developing a standard and objective definition of natriuretic responsiveness.

Our investigation has several limitations. We analyzed a subset of patients from a prospective cohort study performed in the ED. Diuretic dosing was at the discretion of the treating physician and was not standardized; thus, our results could have been influenced by under dosing. In addition, we did not consider other therapies the patient may have received in addition to loop diuretics. Further, the median time from initial ED diuretic dose to the time of urine electrolyte analysis in our study was 8.3 h with an interquartile range of 3–12.9 h. The majority of natriuresis occurs within the first 180 min following parenteral administration of loop diuretics [20], suggesting the majority of natriuresis may have occurred by the time our urine samples were collected and analyzed. However, many of our patients still had urinary sodium measures consistent with optimal daily natriuresis, suggesting these patients had a brisk and appropriate natriuretic response. Our patients did not have a bladder scan prior to diuretic administration and were not required to have an indwelling urinary catheter placed, nor did the majority receive one as part of routine ED practice; therefore, total urinary output documentation may be less accurate. In addition, if patients did have a urinary catheter placed in the ED, their measured urinary sodium at time of collection could be inaccurate secondary to residual urine in their Foley bag. Because this was a retrospective study, we are unable to classify the severity of the AHF exacerbations in our study. Measurements such as home diuretic dose, time from last diuretic dose, and weight gain compared to dry weight would have added valuable information regarding the extent of baseline disease and the severity of the exacerbation that prompted the patient to seek treatment.

## Conclusions

In conclusion, depending on the definition utilized, the proportion of patients identified as diuretic resistant varies anywhere from 6 to 17.1%. This may impact treatment decisions, especially if changes in therapy are made early in a patient’s course based on these definitions, prior to diuretic resistance becoming clinically apparent. The measure most predictive of diuretic resistance in our investigation was urinary sodium <50 meq/L. These patients may have an increase in hospital readmission for AHF and trends toward a longer inpatient LOS. Measuring urine electrolytes and predicting natriuretic response shortly after initial ED treatment present an opportunity to identify patients who may need intensified therapy early in their hospital course to provide adequate decongestion. These findings need to be explored in a larger cohort of ED patients who receive a standardized initial diuretic regimen, continuous urinary measurements over a 6-h time period, and adjust for other confounders that may contribute to LOS and readmission. This will enable us to quantify natriuresis and predict natriuretic responsiveness via urinary electrolyte patterns, determining the earliest time point during an ED presentation that diuretic resistance can be identified.

## Additional files

Additional file 1: Table S1. Descriptive statistics by diuretic resistance assessed using a FeNa <0.2%. (DOC 65 kb)

Additional file 2: Table S2. Descriptive statistics by diuretic resistance assessed using a Na/K ratio < 1. (DOC 64 kb)

## Abbreviations

AHF: Acute heart failure
ED: Emergency department
FeNa: Fractional excretion of sodium
K: Potassium
LOS: Length of stay
Na: Sodium
SBP: Systolic blood pressure
