# Supplementary material for: Markers of diuretic resistance in emergency department patients with acute heart failure
Source: Int J Emerg Med. 2017 May 8;10:17. doi: 10.1186/s12245-017-0143-x (PMC5422212; doi:10.1186/s12245-017-0143-x)
Supplement: Supplementary file 1 — Descriptive statistics by diuretic resistance assessed using a FeNa <0.2%. (DOC 65 kb) [file 12245_2017_143_MOESM1_ESM.doc]

**Additional file 1: Table S1. Descriptive statistics by diuretic resistance assessed using a FeNa<0.2%*.***

|  | **N** | **Normal (N=176)** | | **Diuretic resistant (N=11)** | | **P-value** |
| --- | --- | --- | --- | --- | --- | --- |
| **Age** | 187 | 64 | (55,73) | 66 | (56, 72) | 0.93 |
| **Sex**  Female  Male | 187 | 35%  65% | 61  115 | 45%  55% | 5  6 | 0.47 |
| **Race**  AA  Other | 187 | 51%  49% | 89  87 | 36%  64% | 4  7 | 0.36 |
| **History of Renal Disease**  **No**  **Yes** | 184 | 73%  27% | 126  47 | 100%  0% | 11  0 | 0.05 |
| **Home Diuretic Dose (mg)** | 77 | 60 | (40,80) | 50 | (35,65) | 0.41 |
| **SBP** | 187 | 149 | (130, 179) | 142 | (130, 152) | 0.24 |
| **BUN** | 185 | 22 | (15, 35) | 17 | (14,20) | 0.09 |
| **Serum creatinine (baseline)** | 187 | 1.4 | (1.1, 2.1) | 1.0 | (0.9,1.2) | 0.007 |
| **Serum creatinine (12-24 hours)** | 187 | 1.5 | (1.2, 2.2) | 1.2 | (1.0, 1.2) | 0.005 |
| **Urine creatinine (12-24 hours)** | 187 | 37 | (23, 63) | 151 | (119, 82) | < 0.001 |
| **eGFR** | 187 | 50 | (31, 71) | 63 | (61, 73) | 0.04 |
| **BNP** | 187 | 1240 | (560, 2232) | 894 | (448, 1318) | 0.14 |
| **Urinary sodium (12-24 hours)** | 187 | 92 | (70, 112) | 24 | (20, 27) | < 0.001 |
| **Na/K ratio** | 187 | 4.1 | (2.24, 6.54) | 0.4 | (0.28, 0.52) | < 0.001 |
| **Serum sodium (baseline)** | 187 | 140 | (138, 142) | 139 | (138, 140) | 0.25 |
| **Serum sodium (12-24 hours)** | 187 | 139 | (138, 141) | 139 | (138, 140) | 0.74 |
| **FeNa** | 187 | 2.95 | (1.13, 5.24) | 0.14 | (0.12, 0.17) | < 0.001 |
| **Ejection fraction** | 175 |  |  |  |  | 0.89 |
| Normal (greater than 55 percent) |  | 33% | (54) | 33% | (3) |  |
| Mild (45-55 percent) |  | 14% | (23) | 11% | ( 1) |  |
| Moderate (25-44 percent) |  | 23% | (38) | 33% | ( 3) |  |
| Severe (less than 25 percent) |  | 31% | (51) | 22% | ( 2) |  |
| **ED lasix/furosemide dose categorized** | 159 |  |  |  |  | 0.822 |
| ≥  80 mg |  | 33% | ( 50) | 22% | ( 2) |  |
| <  80 mg |  | 67% | ( 100) | 78% | (7) |  |
| **LOS (days)** | 187 | 4 | (2, 6) | 4 | (2.5, 6) | 0.79 |
| **Urine output up to second visit** | 172 | 1725 | (825, 2850) | 1925 | (1400, 2138) | 0.93 |
| **ED revisit for HF** | 187 |  |  |  |  | 0.382 |
| No |  | 87% | (135) | 81% | ( 26) |  |
| Yes |  | 13% | ( 20) | 19% | (  6) |  |
| **Readmission for HF** | 187 |  |  |  |  | 0.63 |
| No |  | 86% | (151) | 91% | (10) |  |
| Yes |  | 14% | ( 25) | 9% | (1) |  |
| **Status**  Alive  Deceased | 187 | 95%  5% | (168)  (8) | 100%  0% | (11)  (0) | 0.47 |
