# Supplementary material for: Markers of diuretic resistance in emergency department patients with acute heart failure
Source: Int J Emerg Med. 2017 May 8;10:17. doi: 10.1186/s12245-017-0143-x (PMC5422212; doi:10.1186/s12245-017-0143-x)
Supplement: Supplementary file 2 — Descriptive statistics by diuretic resistance assessed using a Na/K ratio < 1. (DOC 64 kb) [file 12245_2017_143_MOESM2_ESM.doc]

**Additional file 2: Table S2. Descriptive statistics by diuretic resistance assessed u**sing a Na/K ratio < 1.

|  | **N** | **Normal (N=167)** | | **Diuretic resistant (N=20)** | | **P-value** |
| --- | --- | --- | --- | --- | --- | --- |
| **Age** | 187 | 63 | (55, 73) | 70 | (62, 78) | 0.07 |
| **Sex**  Female  Male | 187 | 35%  65% | 58  109 | 40%  60% | 8  12 | 0.64 |
| **Race**  AA  Other | 187 | 53%  47% | 88  79 | 25%  75% | 5  15 | 0.02 |
| **History of Renal Disease**  **No**  **Yes** | 184 | 73%  27% | 121  44 | 84%  16% | 16  3 | 0.3 |
| **Home Diuretic Dose (mg)** | 77 | 60 | (40,80) | 70 | (40,85) | 0.73 |
| **SBP** | 187 | 149 | (130, 178) | 141 | (127, 164) | 0.41 |
| **BUN** | 185 | 22 | (15, 35) | 23 | (15, 38) | 0.75 |
| **Serum creatinine (baseline)** | 187 | 1.4 | (1.1, 2.1) | 1.2 | (1.0, 2.0) | 0.19 |
| **Serum creatinine (12-24 hours)** | 187 | 1.5 | (1.1, 2.2) | 1.2 | (1.0, 1.9) | 0.12 |
| **Urine creatinine (12-24 hours)** | 187 | 35 | (22, 60) | 114 | (63, 147) | < 0.001 |
| **eGFR** | 187 | 50 | (32, 72) | 58 | (35, 69) | 0.42 |
| **BNP** | 187 | 1217 | (545, 2170) | 1602 | (783, 2610) | 0.151 |
| **Urinary sodium (12-24 hours)** | 187 | 96 | (72, 113) | 30 | (23,36) | < 0.0011 |
| **Na/K ratio** | 187 | 4.3 | (2.4, 6.7) | 0.5 | (0.34, 0.71) | < 0.001 |
| **Serum sodium (baseline)** | 187 | 140 | (138, 142) | 139 | (137, 140) | 0.05 |
| **Serum sodium (12-24 hours)** | 187 | 139 | (138, 141) | 139 | (138, 141) | 0.151 |
| **FeNa** | 187 | 3.21 | (1.30, 5.48) | 0.20 | (0.15, 0.76) | < 0.001 |
| **Ejection fraction** | 175 |  |  |  |  | 0.632 |
| Normal (greater than 55 percent) |  | 32% | (51) | 33% | (6) |  |
| Mild (45-55 percent) |  | 14% | (22) | 11% | (2) |  |
| Moderate (25-44 percent) |  | 24% | (37) | 22% | (4) |  |
| Severe (less than 25 percent) |  | 30% | (47) | 33% | (6) |  |
| **ED lasix/furosemide dose categorized** | 159 |  |  |  |  | 0.10 |
| ≥  80 mg |  | 30% | ( 43) | 50% | (  9) |  |
| <  80 mg |  | 70% | ( 98) | 50% | ( 17) |  |
| **LOS (days)** | 187 | 4 | (2, 6) | 3.5 | (2.8, 6) | 0.77 |
| **Urine output up to second visit** | 172 | 1750 | (860,2906) | 1825 | (741,2131) | 0.40 |
| **ED revisit for HF** | 187 |  |  |  |  | 0.88 |
| No |  | 86% | (144) | 85% | (17) |  |
| Yes |  | 14% | ( 23) | 15% | (3) |  |
| **Readmission for HF** | 187 |  |  |  |  | 0.95 |
| No |  | 84% | (141) | 85% | (17) |  |
| Yes |  | 16% | ( 26) | 15% | (3) |  |
| **Status**  Alive  Deceased | 187 | 96%  4% | (161)  (6) | 90%  10% | (18)  (2) | 0.18 |
